# Risk of venous thromboembolism in patients with congenital heart disease: a nationwide, register-based, case–control study

**DOI:** 10.1093/ehjopen/oeae089

**Published:** 2024-10-10

**Authors:** Dagmara Cuszynska-Kruk, Maria Fedchenko, Kok Wai Giang, Mikael Dellborg, Peter Eriksson, Per-Olof Hansson, Zacharias Mandalenakis

**Affiliations:** Department of Molecular and Clinical Medicine, Institute of Medicine, Sahlgrenska Academy, University of Gothenburg, Diagnosvägen 11, SE-416 50 Gothenburg, Sweden; Department of Molecular and Clinical Medicine, Institute of Medicine, Sahlgrenska Academy, University of Gothenburg, Diagnosvägen 11, SE-416 50 Gothenburg, Sweden; Department of Medicine, Geriatrics and Emergency Medicine, Region Västra Götaland, Sahlgrenska University Hospital/Östra, Gothenburg, Sweden; Department of Molecular and Clinical Medicine, Institute of Medicine, Sahlgrenska Academy, University of Gothenburg, Diagnosvägen 11, SE-416 50 Gothenburg, Sweden; Department of Medicine, Geriatrics and Emergency Medicine, Region Västra Götaland, Sahlgrenska University Hospital/Östra, Gothenburg, Sweden; Department of Molecular and Clinical Medicine, Institute of Medicine, Sahlgrenska Academy, University of Gothenburg, Diagnosvägen 11, SE-416 50 Gothenburg, Sweden; Department of Medicine, Geriatrics and Emergency Medicine, Region Västra Götaland, Sahlgrenska University Hospital/Östra, Gothenburg, Sweden; Department of Molecular and Clinical Medicine, Institute of Medicine, Sahlgrenska Academy, University of Gothenburg, Diagnosvägen 11, SE-416 50 Gothenburg, Sweden; Department of Medicine, Geriatrics and Emergency Medicine, Region Västra Götaland, Sahlgrenska University Hospital/Östra, Gothenburg, Sweden; Adult Congenital Heart Unit, Department of Medicine, Sahlgrenska University Hospital, Gothenburg, Sweden; Department of Molecular and Clinical Medicine, Institute of Medicine, Sahlgrenska Academy, University of Gothenburg, Diagnosvägen 11, SE-416 50 Gothenburg, Sweden; Department of Medicine, Geriatrics and Emergency Medicine, Region Västra Götaland, Sahlgrenska University Hospital/Östra, Gothenburg, Sweden; Department of Molecular and Clinical Medicine, Institute of Medicine, Sahlgrenska Academy, University of Gothenburg, Diagnosvägen 11, SE-416 50 Gothenburg, Sweden; Department of Medicine, Geriatrics and Emergency Medicine, Region Västra Götaland, Sahlgrenska University Hospital/Östra, Gothenburg, Sweden; Adult Congenital Heart Unit, Department of Medicine, Sahlgrenska University Hospital, Gothenburg, Sweden

**Keywords:** Congenital heart disease, Venous thromboembolism, Register based, Nationwide study

## Abstract

**Aims:**

Patients with congenital heart disease (CHD) have an increased risk of developing acquired cardiovascular diseases. However, the risk of venous thromboembolism (VTE) in patients with CHD is unknown. We aimed to investigate the incidence and risk of VTE in patients with CHD compared with matched controls without CHD.

**Methods and results:**

Data from Swedish health registers were used to identify all patients with CHD between 1970 and 2017 in Sweden. Each patient with CHD was matched with 10 controls from the Swedish Total Population Register. The primary outcome of the study was an event of VTE. Follow-up was from birth until VTE, death, or the end of the study (2017). Cox proportional hazard models were used to investigate the risk of VTE in patients with CHD and controls. A total of 67 814 patients with CHD and 583 709 matched controls were identified and included in the study. During a mean follow-up of 15.9 (SD ± 12.5) years, 554 (0.8%) patients with CHD and 1571 (0.3%) controls developed VTE. The risk of VTE was 3.3 [95% confidence interval [CI] 2.6–3.4) times higher in patients with CHD than in controls. Patients with conotruncal defects had the highest risk of VTE (hazard ratio 7.06, 95% CI 5.52–9.03).

**Conclusion:**

In our nationwide study, we found that the risk of VTE in patients with CHD was more than three times higher than in matched controls. The highest risk of VTE was in patients with the most complex lesions. Further research is crucial to clarify the underlying risk factors and prevent VTE in patients with CHD.

## Structured Graphical abstract

**Graphical Abstract:**
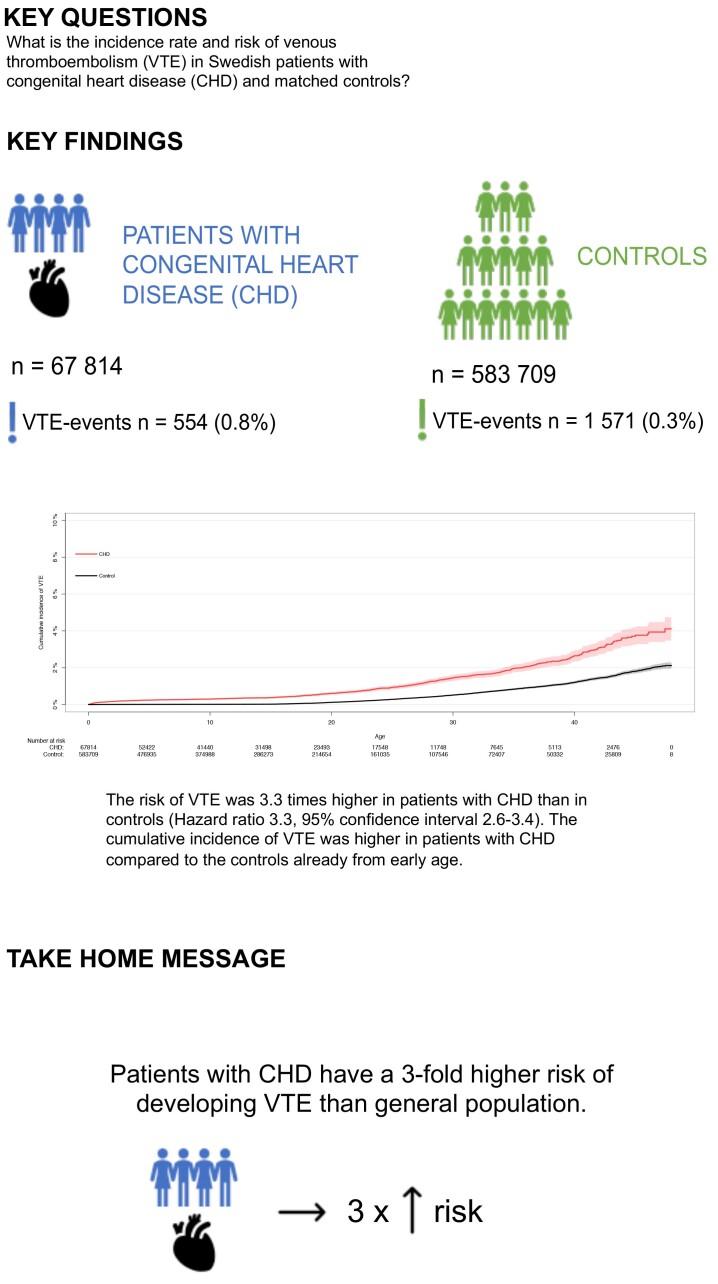

## Introduction

Venous thromboembolism (VTE), including pulmonary embolism (PE) and deep vein thrombosis (DVT), is the third most common vascular disease globally^1^ with an incidence of 100–200 cases per 100 000 person-years.^2–7^ The risk of VTE increases with age.^2,4,7^ The survival of patients with congenital heart disease (CHD) has increased exponentially between 1980 and 2000. Currently, 97% of patients with CHD survive to adult age, although the risk of mortality is still significantly higher in patients with CHD than in the general population.^8^ As patients with CHD age, the risk of conditions related to increased age and acquired risk factors,^9^ such as diabetes mellitus^10^ or hypertension, also increases. However, because of their condition, patients with CHD already have an increased risk of several medical conditions such as atrial fibrillation,^11^ ischaemic stroke,^12,13^ heart failure,^14,15^ and ischaemic heart disease,^16^ in comparison with matched controls. The risk of VTE in patients with CHD in Sweden is unknown, with no previous studies available. The aim of the present study was to determine the incidence rate and risk of VTE in patients with CHD compared with matched controls that do not have CHD in Sweden. An additional aim was to determine if the risk of VTE varies depending on the type of CHD lesion.

## Methods

### Study population and data collection procedures

We used data from the Swedish National Patient Register to identify all patients born in Sweden between 1 January 1970 and 31 December 2017 with a diagnosis of CHD. Ten controls from the Total Population Register in Sweden were randomly assigned to each included patient with CHD and matched by sex and year of birth. The exclusion criteria were country of birth other than Sweden and year of birth between 1930 and1969. The number of controls was chosen to cover an eventual loss of controls owing to the exclusion criteria, moving abroad from Sweden, or other factors that would make follow-up impossible. Because of a potential loss of controls, the ratio between the patients with CHD and controls included in this study might not be 1 to 10. Patients with a diagnosis of CHD after VTE event were not included in the study. Patients with CHD and controls were followed up through the Swedish National Patient Register and the National Cause of Death Register. The Swedish National Patient Register includes data since 1987 for inpatient care and also data from 2001 for outpatient care. The study population has been included and described in earlier studies.^11,12,16,17^

### Definitions

Congenital heart disease and VTE were defined as receiving a corresponding diagnosis according to the International Statistical Classification of Disease and Related Health Problems (ICD) (see Supplementary material online, *Tables S1* and *S2*). Venous thromboembolism is an umbrella term that includes PE, DVT, and thromboembolic event in other veins of the human body. In this study, the events were divided into PE (embolus in the lung), DVT (thrombosis in the deep veins), and other thromboembolic event (thromboembolic event in the remaining veins). The ICD Eighth, Ninth (Swedish version), and Tenth Revisions were used. Patients were divided into six groups according to the hierarchic lesion categorization proposed by Botto *et al.*^18^ and modified by Liu *et al.*^19^ This categorization has been used and described in earlier studies.^11,16,20,21^ The six groups were following: conotruncal defects, non-conotruncal defects, coarctation of the aorta, ventricular septal defect (VSD), atrial septal defect (ASD), and other lesions. Because of a low number of events, the groups were classified into complex lesions and non-complex lesions. The complex lesion group comprised patients with conotruncal and non-conotruncal defects, and the non-complex lesion group included the remaining four groups.

### Statistics

R Studio software (version 4.2.0)^22^ was used for the statistical analyses (The R Project for Statistical Computing, Vienna, Austria). Continuous data are presented as mean and standard deviation (SD) and categorical data as frequency and percentage. For continuous data, the *t*-test was used to compare differences in mean values and the *χ*^2^ test for categorical data. A *P* < 0.05 was recognized as statistically significant. In the current study, all patients with CHD and matched controls were followed from birth until the first event of VTE, death, or the end of the study (31 December 2017), whichever occurred first. The incidence rate per 10 000 person-years was estimated as the total number of events divided by the total follow-up time (in years). Because of the long follow-up period, cumulative incidence curves were estimated with competing risk according to the Fine–Grey method, with 95% confidence intervals (CIs). Hazard ratios (HRs) with 95% CIs were estimated using a Cox regression model to compare the risk of VTE between CHD and controls (reference group). All models were adjusted for sex, year of birth, and baseline comorbidities (defined as before an event of VTE). A sensitivity analysis was conducted to examine how surgery affected the frequency of VTE events.

### Ethics

The project has been approved by the Regional Research Ethics Board in Gothenburg (Gbg 912-16, T619-18). The data used in this study were pseudonymized; therefore, an exception was made regarding informed consent for participation in medical research.

## Results

A total of 67 814 individuals with CHD and 583 709 matched controls were identified. Of these, 34 173 (50.4%) and 294 146 (50.4%) were men, respectively. *Table 1* shows the characteristics of the study populations. During a mean follow-up time of 15.9 (SD ± 12.5) years, 554 (0.8%) patients with CHD and 1571 (0.3%) controls developed VTE; of these, 241 (0.4%) patients with CHD and 1045 (0.2%) controls developed DVT and 162 (0.2%) patients with CHD and 452 (0.1%) controls developed PE.

**Table 1:** Characteristics of the study population

Characteristic	Patients with CHD (*n* = 67 814), *n* (%)	Controls (*n* = 583 709), *n* (%)	*P* value
Sex			1.000
Male	34 173 (50.4%)	294 146 (50.4%)	
Female	33 641 (49.6%)	289 563 (49.6%)	
Year of birth	1999.6 ± 12.8	2000.6 ± 12.6	<0.001
Birth period			<0.001
1970–1979	6760 (10.0%)	51 843 (8.9%)	
1980–1989	8933 (13.2%)	65 254 (11.2%)	
1990–1999	13 054 (19.2%)	109 864 (18.8%)	
2000–2009	20 372 (30.0%)	180 630 (30.9%)	
2010–2017	18 695 (27.6%)	176 118 (30.2%)	
Classification 1			
Lesion group 1	4969 (7.3%)	42 379 (7.3%)	
Lesion group 2	3671 (5.4%)	29 714 (5.1%)	
Lesion group 3	3183 (4.7%)	26 318 (4.5%)	
Lesion group 4	22 072 (32.5%)	191 542 (32.8%)	
Lesion group 5	13 730 (20.2%)	121 941 (20.9%)	
Lesion group 6	20 189 (29.8%)	171 815 (29.4%)	
Classification 2			
Complex lesion	8640 (12.7%)	72 093 (12.4%)	
Non-complex lesion	59 174 (87.3%)	511 616 (87.6%)	
Follow-up time	15.9 ± 12.5	16.9 ± 12.5	<0.001
VTE event	554 (0.8%)	1571 (0.3%)	<0.001
DVT event	241 (0.4%)	1045 (0.2%)	<0.001
PE event	162 (0.2%)	452 (0.1%)	<0.001
Other thromboembolic event	234 (0.3%)	348 (0.1%)	<0.001

Lesion group 1—conotruncal defects, lesion group 2—non-conotruncal defects, lesion group 3—coarctation of the aorta, lesion group 4—ventricular septal defect, lesion group 5—atrial septal defect, lesion group 6—other lesions. Complex lesions consist of Groups 1 and 2. Non-complex lesions consist of Groups 3–6. Controls did not have any congenital heart defects. Numbers in lesion groups in the control column show the number of matched controls in each lesion group. Year of birth and follow-up time in the table are presented as mean with SD.

CHD, congenital heart defects; VTE, venous thromboembolism; DVT, deep vein thrombosis; PE, pulmonary embolism.

Characteristics of the study population with CHD according to lesion group are shown in *Table 2*. The highest number of VTE events was found in lesion group 1 (101 events, 2.0%). Dividing VTE events into DVT, PE, and other thromboembolic event showed that all three types of thromboembolic events were more common in patients with CHD than in their controls (*Table 3*). The number of VTE events in patients with complex lesions was 160 (1.9%) and 394 in patients with non-complex lesions (0.7%). The percentage of events in the DVT, PE, and other thromboembolic event groups were higher in patients with complex lesions than in those with non-complex lesions. Deep vein thrombosis was diagnosed in 51 (0.6%) patients in the complex group and 190 (0.3%) in patients with non-complex lesions. For PE, the numbers were 46 (0.5%) and 116 (0.2%), respectively.

**Table 2:** Characteristics of patients with congenital heart defects, according to lesion group

Patients with CHD							
Characteristic	Lesion group 1	Lesion group 2	Lesion group 3	Lesion group 4	Lesion group 5	Lesion group 6	*P* value
Sex							<0.001
Male	2953 (59.4%)	1784 (48.6%)	1917 (60.2%)	10 480 (47.5%)	6549 (47.7%)	10 490 (52.0%)	
Female	2016 (40.6%)	1887 (51.4%)	1266 (39.8%)	11 592 (52.5%)	7181 (52.3%)	9699 (48.0%)	
Year of birth	1995.0 ± 12.9	1992.9 ± 13.2	1995.0 ± 13.6	2003.1 ± 11.3	2002.8 ± 12.0	1996.6 ± 12.9	<0.001
Birth period							<0.001
1970–1979	752 (15.1%)	726 (19.8%)	544 (17.1%)	1196 (5.4%)	1018 (7.4%)	2524 (12.5%)	
1980–1989	990 (19.9%)	784 (21.4%)	599 (18.8%)	1861 (8.4%)	1060 (7.7%)	3639 (18.0%)	
1990–1999	1221 (24.6%)	905 (24.7%)	690 (21.7%)	3220 (14.6%)	1922 (14.0%)	5096 (25.2%)	
2000–2009	1180 (23.7%)	770 (21.0%)	760 (23.9%)	8107 (36.7%)	4816 (35.1%)	4739 (23.5%)	
2010–2017	826 (16.6%)	486 (13.2%)	590 (18.5%)	7688 (34.8%)	4914 (35.8%)	4191 (20.8%)	
Follow-up time in years	16.5 ± 13.2	13.9 ± 14.1	19.6 ± 13.9	13.9 ± 11.0	14.4 ± 11.9	18.8 ± 13.2	<0.001
VTE event	101 (2.0%)	59 (1.6%)	25 (0.8%)	86 (0.4%)	102 (0.7%)	181 (0.9%)	<0.001

Lesion group 1—conotruncal defects, lesion group 2—non-conotruncal defects, lesion group 3—coarctation of the aorta, lesion group 4—ventricular septal defect, lesion group 5—atrial septal defect, lesion group 6—other lesions. Year of birth and follow-up time in the table are presented as mean with SD.

CHD, congenital heart defects; VTE, venous thromboembolism; PE, pulmonary embolism.

**Table 3:** Number of events of venous thromboembolism in patients with congenital heart defects and controls divided by lesion group

	Complex lesion			Non-complex lesion		
	Patients with CHD, *n* (%)	Controls, *n* (%)	*P* value	Patients with CHD, *n* (%)	Controls, *n* (%)	*P* value
VTE event	160 (1.9)	318 (0.4)	<0.001	394 (0.7)	1253 (0.2)	<0.001
DVT event	51 (0.6)	206 (0.3)	<0.001	190 (0.3)	839 (0.2)	<0.001
PE event	46 (0.5)	93 (0.1)	<0.001	116 (0.2)	359 (0.1)	<0.001
Other thromboembolic event	86 (1.0)	66 (0.1)	<0.001	148 (0.3)	282 (0.1)	<0.001

Complex lesions consist of conotruncal and non-conotruncal defects. Non-complex lesions consist of coarctation of the aorta, ventricular septal defect, atrial septal defect, and other lesions. Other thrombosis event indicates thrombosis in veins other than the deep veins of the body or pulmonary embolism.

CHD, congenital heart defects; VTE, venous thromboembolism; DVT, deep vein thrombosis; PE, pulmonary embolism.

As shown in *Table 4*, the incidence of VTE per 10 000 patient-years at risk in patients with CHD was 5.13 (95% CI 4.71–5.57), compared with 1.59 (95% CI 1.51–1.67) in the control group. The incidence was higher in women than in men in both the CHD and control groups. In all lesion groups, the incidence of VTE was higher among individuals with CHD than that in their controls. The incidence was highest in patients from lesion group 1 with conotruncal defects. Generally, the incidence of a VTE event increased with older age.

**Table 4:** Incidence of venous thromboembolism in patients with congenital heart disease and matched controls per 10 000 patient-years at risk

	Patients with CHD			Controls		
	Patients with CHD, *n* (%)	VTE events, *n* (%)	Incidence rate of VTE (95% CI)	Controls, *n* (%)	VTE events, *n* (%)	Incidence rate of VTE (95% CI)
All CHD	67 814 (100)	554 (0.82)	5.13 (4.71–5.57)	583 709 (100)	1571 (0.27)	1.59 (1.51–1.67)
Lesion group 1	4969 (7.3)	101 (2.03)	12.35 (10.06–15.01)	42 379 (7.3)	171 (0.40)	1.91 (1.63–2.22)
Lesion group 2	3671 (5.4)	59 (1.61)	11.56 (8.80–14.91)	29 714 (5.1)	147 (0.49)	2.14 (1.80–2.51)
Lesion group 3	3183 (4.7)	25 (0.79)	4.01 (2.59–5.92)	26 318 (4.5)	108 (0.41)	1.95 (1.60–2.36)
Lesion group 4	22 072 (32.5)	86 (0.39)	2.80 (2.24–3.46)	191 542 (32.8)	341 (0.18)	1.30 (1.17–1.45)
Lesion group 5	13 730 (20.2)	102 (0.74)	5.15 (4.20–6.26)	121 941 (20.9)	236 (0.19)	1.36 (1.20–1.55)
Lesion group 6	20 189 (29.8)	181 (0.90)	4.76 (4.09–5.51)	171 815 (29.4)	568 (0.33)	1.68 (1.54–1.82)
Complex lesion	8640 (12.7)	160 (1.85)	12.05 (10.25–14.06)	72 093 (12.4)	318 (0.44)	2.01 (1.79–2.24)
Non-complex lesion	59 174 (87.3)	394 (0.67)	4.16 (3.76–4.59)	511 616 (87.6)	1253 (0.24)	1.51 (1.43–1.60)
1970–1979	6760 (10.0)	199 (2.94)	8.58 (7.43–9.86)	51 843 (8.9)	712 (1.37)	3.24 (3.00–3.49)
1980–1989	8933 (13.2)	145 (1.62)	5.96 (5.03–7.01)	65 254 (11.2)	471 (0.72)	2.25 (2.05–2.46)
1990–1999	13 054 (19.2)	98 (0.75)	3.56 (2.89–4.34)	109 864 (18.8)	310 (0.28)	1.22 (1.09–1.36)
2000–2009	20 372 (30.9)	60 (0.29)	2.38 (1.82–3.06)	180 630 (30.9)	44 (0.02)	0.19 (0.14–0.26)
2010–2017	18 695 (27.6)	52 (0.28)	6.70 (5.00–8.78)	176 118 (30.2)	34 (0.02)	0.46 (0.32–0.64)
Women	33 641 (49.6)	295 (0.88)	5.54 (4.93–6.21)	289 563 (49.6)	937 (0.32)	1.92 (1.80–2.05)
Men	34 173 (50.4)	259 (0.76)	4.73 (4.17–5.34)	294 146 (50.4)	634 (0.22)	1.27 (1.17–1.37)

Incidence rate is per 10 000 patient-years at risk. Lesion group 1—conotruncal defects, lesion group 2—non-conotruncal defects, lesion group 3—coarctation of the aorta, lesion group 4—ventricular septal defect, lesion group 5—atrial septal defect, lesion group 6—other lesions. Complex lesions consist of Groups 1 and 2. Non-complex lesions consist of Groups 3–6.

CHD, congenital heart defects; VTE, venous thromboembolism; CI, confidence interval.

Regardless of group, all patients with CHD had higher risk of VTE than their controls (*Table 5*). The risk was highest in lesion group 1 (conotruncal defects), lesion group 2 (non-conotruncal defects), and lesion group 5 (ASD): HR 7.06 (95% CI 5.52–9.03), 5.86 (95% CI 4.33–7.94), and 3.74 (95% CI 2.97–4.72), respectively. The highest risk of VTE was in patients with CHD born between 2010 and 2017, with HR 14.54 (95% CI 9.44–22.40). For all birth periods before 2000, the risk was not >2.94 compared with their controls.

**Table 5:** Risk of venous thromboembolism in patients with congenital heart disease and matched controls

	VTE events in patients with CHD, *n*	VTE events in controls, *n*	HR (95% CI)
All CHD	554	1571	3.30 (2.99–6.63)
Lesion group 1	101	171	7.06 (5.52–9.03)
Lesion group 2	59	147	5.86 (4.33–7.94)
Lesion group 3	25	108	2.09 (1.35–3.23)
Lesion group 4	86	341	2.09 (1.65–2.65)
Lesion group 5	102	236	3.74 (2.97–4.72)
Lesion group 6	181	568	2.85 (2.41–3.36)
Complex lesion	160	318	6.55 (5.42–7.93)
Non-complex lesion	394	1253	2.74 (2.45–3.07)
1970–1979	199	712	2.74 (2.34–3.21)
1980–1989	145	471	2.68 (2.23–3.23)
1990–1999	98	310	2.94 (2.35–3.69)
2000–2009	60	44	12.38 (8.39–18.26)
2010–2017	52	34	14.54 (9.44–22.40)
Women	295	937	2.96 (2.59–3.37)
Men	259	634	3.81 (3.29–4.40)

Lesion group 1—conotruncal defects, lesion group 2—non-conotruncal defects, lesion group 3—coarctation of the aorta, lesion group 4—ventricular septal defect, lesion group 5—atrial septal defect, lesion group 6—other lesions. Complex lesions consist of Groups 1 and 2. Non-complex lesions consist of Groups 3–6.

VTE, venous thromboembolism; CHD, congenital heart defects; HR, hazard ratio; CI, confidence interval.

**Figure 1:**
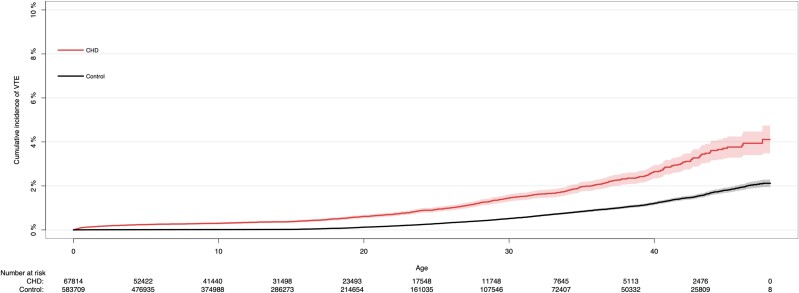
Cumulative incidence of venous thromboembolism (%) in patients with congenital heart defects (red) and controls (black) at age 0–47 years. VTE, venous thromboembolism; CHD, congenital heart disease.

The cumulative incidence of VTE for patients with CHD and controls at age 0–47 years is shown in *Figure 1*. From an early age, patients with CHD showed a higher cumulative incidence of VTE than controls. The cumulative incidence increased most at ∼40 years of age for both patients with CHD and controls.

*Table 6* lists comorbidities and history of surgery in the study populations before a VTE event. Overall, heart surgery 6 months before an event was more common than any of the comorbidities in patients with CHD (0.3%). The most common comorbidity in the control population was hypertension, with 28 cases (<0.01%).

**Table 6:** Comorbidities and history of surgery

Comorbidity or surgery	Patients with CHD, *n* (%)	Controls, *n* (%)	*P* value
Hypertension	17 (<0.01%)	28 (<0.01%)	<1.000
Diabetes mellitus	10 (<0.01%)	23 (<0.01%)	0.001
Atrial fibrillation	27 (<0.01%)	4 (<0.01%)	<0.001
Ischaemic stroke	17 (<0.01%)	5 (<0.01%)	<0.001
Hyperlipidaemia	6 (<0.01%)	9 (<0.01%)	0.001
Heart surgery within 6 months	177 (0.3%)	2 (<0.01%)	<0.001
Previous heart surgery	15 012 (22.1%)	110 (<0.01%)	<0.001
Cancer	28 (<0.01%)	85 (<0.01%)	<0.001

Heart surgery within 6 months is defined as heart surgery 6 months prior to a venous thrombosis event. Previous heart surgery is defined as heart surgery prior to a venous thrombosis event.

CHD, congenital heart defect.

In a sensitivity analysis where all patients with prior thoracic surgery at any time before a VTE event were excluded, there were 26 (0.9%) VTE events in the complex lesion group and 315 (0.4%) events in their controls. In the non-complex lesion group, the number of events in cases and controls was 286 (0.6%) and 1244 (0.2%), respectively. The risk of VTE in patients with complex CHD lesions was HR 4.25 (95% CI 2.85–6.35) and HR 2.89 (95% CI 2.54–3.28) in patients with non-complex lesions.

## Discussion

A main finding of the present study was that patients with CHD had a more than three times higher risk of developing a VTE compared with matched controls that did not have CHD. The highest risk was in the group with the most complex CHD lesions (conotruncal defects). As expected, the VTE incidence increased with age and was highest in the group born during 1970–1979. The most common comorbidity for patients with CHD was heart surgery.

In a national register study of DVT and PE in Sweden, the expected incidence of VTE in the Swedish population under 40 years of age was similar to the incidence for controls in this study. The incidence rate per 100 000 was 11.7 for PE and 18.2 for DVT in age group 30–39 years.^2^ That study also showed that cancer was the most frequent comorbidity in cases of VTE.^2^ Previous studies have shown that cancer is more common among individuals with CHD than in controls without CHD.^23^ It is important to understand how the prevalence of risk factors for VTE differs between patients with CHD and the control population so that conclusions can be drawn regarding the greatest risk factors for VTE in patients with CHD.

Patients with ASD in this study had the third highest incidence and risk of VTE, and this was higher than in groups with more severe lesions, such as coarctation of the aorta or VSD. This finding is important because ASD is the most common CHD among adults in Sweden.^24^ Individuals with CHD have a high risk of atrial fibrillation,^11^ which affects blood flow through the heart and can lead to the formation of thrombus. In a study on thrombotic events in children with cardiac pathology, 6 of 21 affected children had ASD.^25^ However, all these individuals also had other heart defects. According to the hierarchical classification used in this research, these children would have been categorized into a more severe lesion group than ASD.

Another interesting finding was that the risk of VTE was much higher in the youngest birth cohorts of patients with CHD, in comparison with their controls, with an HR of >12 and 14 in patients with CHD born during 2000–2009 and 2010–2017, respectively. The incidence of VTE increases with age, and the number of events in the control group for the birth periods 2000–2009 and 2010–2017 was low, as expected. However, the high HRs for these birth periods showed that children with CHD have a greater risk of developing VTE (compared with their controls) than adults according to our results. There may be an explanation for this, such as improved diagnostics with wider use of computed tomography. A register study from Canada showed that 96% of children with VTE had a condition associated with thromboembolism.^26^ Because 96% of children had a risk factor for VTE, the percentage of idiopathic events in children can be assumed to be low; therefore, it can be assumed that having a heart defect is a stronger risk factor for thromboembolism during childhood than in adulthood.

In the present study, women in both the CHD and control groups had a higher incidence of VTE than men. This finding is consistent with previous observations of VTE. The study population in the present research was young and of reproductive age. Venous thromboembolism is more common in women than in men.^6,27^ However, the incidence was higher in women with CHD than that in their controls, with a 2.96 times higher risk. For men with CHD, the risk was 3.81 times higher than that in their controls. A possible explanation for these findings might be that in the general population in young adulthood, men have a lower incidence of VTE than women.

In the CALF study, which included patients with Fontan circulation, 10 individuals reported DVT and 10 reported PE, out of the 159 patients in the study population,^28^ which is equal to 12.6%. Patients with Fontan circulation could be compared with our patients who had CHD with a complex lesion; our patients had a percentage of VTE events of 1.85%, which is below the percentage in the CALF study. A possible explanation for the different ages of the populations in the two studies could be that all patients included in the CALF study were over 18 years of age and the mean age was 30.6 years. In the present study, the mean age (based on calculations of the mean patient birth year) was ∼17.4 years. Because VTE is more common with older age, the age difference could explain the different percentage of VTE events in the two studies. Furthermore, patients in the CALF study were selected from among patients in specialized adult congenital heart centres, which could affect the patient sample.

A VTE event occurred in 177 patients with heart surgery prior to the event. Sensitivity analysis showed that patients with CHD who did not undergo surgery had a higher risk of VTE than their controls. The conclusion could be that surgery itself does not increase the risk of a VTE event in patients with CHD, but rather other factors are responsible for this increased risk.

The present study has several strengths, as well as some limitations. The study was conducted among a large population of patients with CHD and controls, but the data lack information about patients born outside Sweden, which means that the study does not reflect the entire Swedish population. The group of CHD is heterogenous and consists of around 1800 different diagnosis combinations according to the European classification. We have used the Botto system due to previous work by us and others. The limitation of our study is that detailed information from medical charts is lacking making further subdivisions into specific diagnosis less meaningful but have the strength of being population based and with a minimum of lost to follow-up over a long period of time. Because VTE becomes more common with age, the younger population in this study could have had an impact on the low number of events. It is likely that an older study population would have had a higher number of events and a higher absolute risk. Furthermore, a limitation is that the Outpatient Register only includes data from 2001 onwards and that the Inpatient Register did not cover the whole of Sweden until 1987 meaning that possible VTE diagnoses before 2001 respectively 1987 could have been missed and the real incidence of VTE and the risk could have been underestimated in this study. Beginning in 1970, data collection for the Inpatient Register went from a pilot project to an all-inclusive effort to cover the entire country in 1987. The present study lack data from primary health care that would probably not affect the number of PE as those patients are usually hospitalized, but the number of DVT events could have been underestimated. A limitation in a register-based study is that the data are not collected by the researchers and in different hospitals, which could lead to differences in diagnoses and usage of ICD codes. To circumvent this limitation, the definition of VTE in this study included thromboses of other and unspecified sites. Another limitation in register-based studies could be low coverage, but because there is a legal obligation to fill in the registers the data were collected from, we can assume that this is not the case in this study. In Sweden, hospital reimbursements are not driven by the ICD code. We cannot rule out registration bias completely, but external validation of the registry shows positive value of 85–95% and sensitivity above 90%.^29^ The possible anticoagulation therapy among the patients and controls was not studied, but the presence of this could have had impact on the results. Despite this limitation, the study findings showed that individuals with CHD are at greater risk of developing VTE compared with a control population.

## Conclusions

In this nationwide, register-based cohort study, the absolute risk of developing VTE was low for both patients with CHD and controls; however, the relative risk was more than three times higher in patients with CHD than in matched controls. The highest incidence and risk of VTE were found among patients in the group with the most severe CHD lesions (conotruncal defects). However, further research on the risk factors is of great importance in patients with CHD for optimization of prophylaxis against VTE.

## Lead author biography


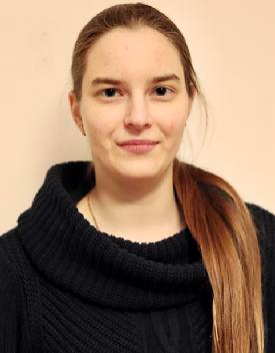


Dagmara Cuszynska-Kruk received her MD in 2023 when she graduated from Sahlgrenska Academy, Gothenburg, Sweden. She is now undertaking the Foundation Program and in her spare time working on the topic of congenital heart disease and venous thromboembolism with a research group led by Zacharias Mandalenakis, MD, PhD, at the Department of Molecular and Clinical Medicine, Sahlgrenska Academy, Gothenburg, Sweden.

## Supplementary Material

oeae089_Supplementary_Data

## Data Availability

Data are available from the sources stated in the article on request to the data providers, fulfilling legal and regulatory requirements, and with permission from the Swedish Ethical Review Authority.
