# Supplementary material for: Risk of venous thromboembolism in patients with congenital heart disease: a nationwide, register-based, case–control study
Source: Eur Heart J Open. 2024 Oct 10;4(6):oeae089. doi: 10.1093/ehjopen/oeae089 (PMC11529300; doi:10.1093/ehjopen/oeae089)
Supplement: oeae089_Supplementary_Data [file oeae089_supplementary_data.pdf]

Supplementary Material

Supplementary Table 1: ICD-codes used for identification of cases of venous thromboembolism

Diagnosis	ICD 8	ICD 9	ICD 10	Event-group
Pulmonary embolism with acute cor pulmonale			I26.0	PE
Pulmonary embolism without acute cor pulmonale	450.01<br>450.02<br>450.03<br>450.09	415B	I26.9	PE
Phlebitis and thrombophlebitis of femoral vein	451.00	451B	I80.1	DVT
Phlebitis and thrombophlebitis of other and unspecified deep vessels of lower extremities	451.00	451B	I80.2	DVT
Phlebitis and thrombophlebitis of lower extremities, unspecified	451.00	451C	I80.3	DVT
Phlebitis and thrombophlebitis of other sites		451W	I80.8	DVT
Phlebitis and thrombophlebitis of unspecified site	451.98<br>451.99	451X	I80.9	DVT
Thrombophlebitis migrans	453.00	453B	I82.1	Other
Embolism and thrombosis of vena cava		453C	I82.2	Other
Embolism and thrombosis of renal vein		453D	I82.3	Other
Embolism and thrombosis of other specified veins		453W	I82.8<br>I82.8W	Other
Embolism and thrombosis of mesenteric vein			I82.8A	Other
Embolism and thrombosis of unspecified vein	453.09	453X	I82.9	Other

PE indicates pulmonary embolism; DVT, deep vein thrombosis. Event-group indicates one of the three event groups investigated in this study.

Supplementary Table 2: ICD-codes used for identification of cases of congenital heart defects and the hierarchical anatomical lesion group that they belong to.

Diagnosis	ICD 8	ICD 9	ICD 10	Lesion group
Common arterial trunk	746.09	745A	Q200	1
Transposition of the great arteries	746.19	745B	Q203	1
Tetralogy of Fallot	746.29	745C	Q213	1
Ventricular septal defect	746.39	745E	Q210	4
Atrial septal defect/patent foramen ovale	746.42	745F	Q211	5
Congenital tricuspid stenosis or atresia	746.54	746B	Q224	6
Ebstein's anomaly	746.54	746C	Q225	6
Congenital stenosis of the aortic valve	746.73	746D	Q230	6
Congenital insufficiency of the aortic valve	746.79	746E	Q231	6
Congenital mitral stenosis	746.59	746F	Q232	6
Congenital mitral insufficiency	746.59	746G	Q233	6
Hypoplastic left heart syndrome	746.74	746H	Q234	2
Congenital subaortic stenosis	746.79	746W	Q244	6
Cor triatriatum	746.89	746W	Q242	6
Infundibular pulmonic stenosis	746.63	746W	Q243	6
Congenital coronary artery anomalies	747.69	746W	Q245	6
Congenital heart block	746.89	746W	Q246	6
Coarctation of the aorta	747.19	747B	Q251	3
Interruption of the aortic arch	747.19	747B	Q252<br>Q253	6
Other unspecified congenital malformations of the aorta	747.29	747C	Q254<br>Q258<br>Q259	6
Congenital malformations of the pulmonary artery	747.34<br>747.39	747D	Q255<br>Q256	6

			Q257	
Congenital malformations of the great veins	747.49<br>747.59	747E	Q260<br>Q261<br>Q262<br>Q263<br>Q264	6
Cor biloculare	746.89	745H	Q208	6
Double outlet right ventricle	746.19	745B	Q201	1
Double outlet left ventricle	746.19	745B	Q202	1
Double inlet ventricle	746.37	745D	Q204	2
Congenitally corrected transposition/discordant atrioventricular and ventriculoatrial connection	746.19	745B	Q205	1
Isomerism of atrial appendages	746.89	745W	Q206	6
Unspecified congenital malformations of the cardiac chambers	746.89	746X	Q208<br>Q209	6
Atrioventricular septal defect	746.47<br>746.46<br>746.43	745G	Q212	2
Aortopulmonary septum defect	746.09	745A	Q214	1
Other congenital malformations of the cardiac septum	746.89	745W	Q218	6
Unspecified congenital malformations of the cardiac septum	746.99	745X	Q219	6
Pulmonary valve atresia	746.64	746A	Q220	6
Congenital stenosis of the pulmonary valve	746.63	746A	Q221	6
Congenital pulmonary valve insufficiency	746.69	746A	Q222	6
Other congenital malformations of the pulmonary valve	746.69	746A	Q223	6

Hypoplastic right heart syndrome	746.69	746B	Q226	6
Other congenital malformations of the tricuspid valve	746.54	746B	Q228<br>Q229	6
Other congenital malformations of aortic and mitral valves	746.89	746W	Q238<br>Q239	6
Other specified congenital malformations of the heart	746.89	746W	Q248	6
Unspecified congenital malformations of the heart	746.99	746X	Q249	6
Patent ductus arteriosus	747.09	747A	Q250	6

Lesion group indicates lesion group of the congenital heart defect according to a hierarchical anatomical classification in which lesion group 1 is conotruncal defects; lesion group 2, non-conotruncal defects; lesion group 3, coarctation of the aorta; lesion group 4, ventricular septal defect; lesion group 5, atrial septal defect and lesion group 6, other heart defects.
